# Construction of an MRI-based decision tree to differentiate autoimmune and autoinflammatory inner ear disease from chronic otitis media with sensorineural hearing loss

**DOI:** 10.1038/s41598-021-98557-w

**Published:** 2021-09-27

**Authors:** Boeun Lee, Yun Jung Bae, Byung Yoon Choi, Young Seok Kim, Jin Hee Han, Hyojin Kim, Byung Se Choi, Jae Hyoung Kim

**Affiliations:** 1 Department of Radiology, College of Medicine, Ewha Womans University, Ewha Womans University Seoul Hospital, 260, Gonghang-daero, Gangseo-gu, Seoul, 07804 Republic of Korea; 2 Department of Radiology, Seoul National University College of Medicine, Seoul National University Bundang Hospital, 82, Gumi-ro 173 Beon-gil, Bundang-gu, Seongnam, 13620 Republic of Korea; 3 Department of Otolaryngology, Seoul National University College of Medicine, Seoul National University Bundang Hospital, 82, Gumi-ro 173 Beon-gil, Bundang-gu, Seongnam, 13620 Republic of Korea; 4 Department of Pathology, Seoul National University College of Medicine, Seoul National University Bundang Hospital, 82, Gumi-ro 173 Beon-gil, Bundang-gu, Seongnam, Republic of Korea

**Keywords:** Immunology, Diseases

## Abstract

Autoimmune and autoinflammatory inner ear diseases (AIED/AID) are characterized by the symptom of sensorineural hearing loss (SNHL). To date, standardized diagnostic tools for AIED/AID are lacking, and clinically differentiating AIED/AID from chronic otitis media (COM) with SNHL is challenging. This retrospective study aimed to construct a magnetic resonance imaging (MRI)-based decision tree using classification and regression tree (CART) analysis to distinguish AIED/AID from COM. In total, 67 patients were enrolled between January 2004 and October 2019, comprising AIED/AID (n = 18), COM (n = 24), and control groups (n = 25). All patients underwent 3 T temporal bone MRI, including post-contrast T1-weighted images (postT1WI) and post-contrast FLAIR images (postFLAIR). Two radiologists evaluated the presence of otomastoid effusion and inner ear contrast-enhancement on MRI. A CART decision tree model was constructed using MRI features to differentiate AIED/AID from COM and control groups, and diagnostic performance was analyzed. High-intensity bilateral effusion (61.1%) and inner ear enhancement (postFLAIR, 93.8%; postT1WI, 61.1%) were the most common findings in the AIED/AID group. We constructed two CART decision tree models; the first used effusion amount as the first partitioning node and postT1WI-inner ear enhancement as the second node, whereas the second comprised two partitioning nodes with the degree of postFLAIR-enhancement of the inner ear. The first and second models enabled distinction of AIED/AID from COM with high specificity (100% and 94.3%, respectively). The amount of effusion and the degree of inner ear enhancement on MRI may facilitate the distinction between AIED/AID and COM with SNHL using decision tree models, thereby contributing to early diagnosis and intervention.

## Introduction

Autoimmune inner ear disease (AIED) is a rare clinical condition characterized by progressive, bilateral, and often asymmetric sensorineural hearing loss (SNHL)^1^. In 15–30% of cases, AIED is secondary to systemic autoimmune diseases, including granulomatosis with polyangiitis (GPA)—previously termed as Wegener’s granulomatosis, Cogan’s syndrome, systemic lupus erythematosus, or Sjögren’s syndrome^1,2^. Autoinflammatory inner ear disease (AID) is a family of rare immune-mediated diseases. Conditions representing AID include neonatal onset multisystem inflammatory disease (NOMID), chronic infantile neurological, cutaneous, and articular (CINCA) syndrome, and Bechet’s disease^3,4^. Although the pathophysiologic features of AIED and AID are distinct, these conditions share similar clinical symptoms, such as bilateral SNHL^3^.

To date, standardized diagnostic tools for the diagnosis of AIED and AID (AIED/AID) are lacking. Currently, immune-mediated inner ear disease manifesting as cochleovestibular dysfunction is diagnosed by exclusion, depending upon clinical symptoms, laboratory tests, and/or steroid responsiveness. In this regard, distinguishing AIED/AID from chronic otitis media (COM) can be challenging due to considerable overlap in clinical features, especially when COM presents with infectious labyrinthitis and SNHL. The treatment for AIED/AID varies; cochlear dysfunction can occasionally be reversed by timely and appropriate medications in the case of GPA (a subset of AIED)^5^, but COM requires surgical treatment, and cochlear dysfunction is often irreversible. Therefore, correct and timely diagnosis is critical for these disease entities. Nevertheless, serological and molecular genetic tests that enable definitive diagnosis are not always feasible in clinical settings, and may not provide a conclusive diagnosis. Thus, in patients with SNHL and suspected AIED/AID who require diagnosis by exclusion, radiologic evaluation using magnetic resonance imaging (MRI) is mandatory to evaluate inner ear features. Nevertheless, there is a paucity of case reports on inner ear MRI findings in AIED/AID due to its low prevalence^6–8^.

In this study, we aimed to evaluate inner ear findings using high-resolution 3 T MRI in patients clinically diagnosed with AIED/AID and to harness MRI findings to accurately differentiate between AIED/AID and COM by adopting classification and regression tree (CART) analysis. CART analysis is a form of binary recursive partitioning that employs non-parametric statistical methods^9^. CART models facilitate differential diagnosis from ease of handling variables and simple interpretation in clinical practice^10^. The purpose of our study was to construct a decision tree based on MRI features in order to distinguish AIED/AID from COM with SNHL.

## Methods

### Ethics approval

The Institutional Review Board of Seoul National University Bundang Hospital approved this retrospective study (No. B-2003-598-101) in accordance with the ethical standards of the institutional research committee and with the Declaration of Helsinki and its later amendments. The requirement for informed consent was waived by the board for all the age groups.

### Study population

We searched the database in our tertiary referral institution for patients who were diagnosed with AIED/AID and received treatment between January 2004 and October 2019. Patients who received a confirmed diagnosis of AIED/AID via serologic, genetic, and/or pathologic tests and underwent high-resolution temporal bone MRI on 3 T were included in the AIED/AID group. The clinical diagnosis of AIED/AID was made based on previously reported criteria as follows. The diagnosis of GPA was based on clinical examination, serologic tests for anti-neutrophil cytoplasmic autoantibodies (ANCA), and/or histopathologic studies^11–16^. The diagnosis of CINCA syndrome/DFNA34 was based on characteristic clinical features, such as a persistent urticarial rash concomitant with systemic inflammation signs and/or molecular analysis of the *NLRP3* gene^17–19^. The diagnosis of Cogan’s syndrome was made solely based on clinical findings such as bilateral ocular and vestibuloauditory symptoms without definite or specific diagnostic tests^20^. Patients who did not undergo temporal bone MRI, had motion or metallic artifacts on MRI, exhibited labyrinthitis ossificans on MRI and/or concurrent temporal bone computed tomography, and/or presented with systemic or temporal bone pathology other than AIED/AID were excluded. In particular, we paid special attention to rule out Meniere’s disease, since its underlying pathogenesis of autoimmunity could be overlapped with AIED/AID. Accordingly, patients who were diagnosed with Meniere’s disease based on the diagnostic criteria were excluded from our study^21^. In addition, patients who presented vestibular symptom alone without auditory symptom were excluded as well.

Patients who underwent mastoidectomy for COM and preoperative high-resolution temporal bone MRI due to SNHL during the same study period were included in the COM group. The control group was comprised of patients who underwent high-resolution temporal bone MRI due to non-cochleovestibular symptoms such as hemifacial spasm, trigeminal neuralgia, non-specific dizziness, presbycusis, or any other symptomatology that was underpinned by etiology other than inner ear pathology.

Audiogram profiles from subjects with COM, GPA, CINCA syndrome/DFNA34, and Cogan syndrome were obtained and compared against each other.

### MRI protocol

Temporal bone MRI was acquired using a 3 T scanner (Achieva and Ingenia, Philips Healthcare, Best, the Netherlands) with a 16- or 32-channel SENSE head coil (Philips Healthcare). We acquired three-dimensional (3D) heavily T2-weighted images (T2WI), two-dimensional (2D) pre-contrast T1-weighted images (T1WI), 3D pre-contrast fluid-attenuation inversion recovery images (preFLAIR), 3D post-contrast T1WI (postT1WI), and 3D post-contrast FLAIR images (postFLAIR). For contrast-enhancement, 0.1 mmol/kg of gadobutrol (Gadovist, Bayer Schering Pharma AG, Berlin, Germany) was intravenously injected as a bolus. PostT1WI and postFLAIR were scanned approximately 2 min and 7 min post-injection, respectively. Whole-brain T2WI and FLAIR were obtained in all patients to exclude other brain pathologies. Detailed parameters of each sequence are summarized in Table S1.

### MRI analyses

Two neuroradiologists (Y.J.B. and B.S.C. with 11 and 21 years of experience, respectively) independently reviewed all temporal bone MRI scans. The radiologists were blinded to the clinical diagnosis at the time of review. After independent evaluation, both radiologists reviewed the MRI scans by consensus and resolved any discrepancies. Based on previous reports of MRI findings in AIED/AID and COM with infectious labyrinthitis^22^, the following MRI features were evaluated: (1) presence, laterality, and amount of mastoid and middle ear effusion (Fig. S1); (2) presence and laterality of any MRI abnormalities in inner ear structures including the cochlea, vestibule, and semicircular canals including (2-1) presence of hyperintensity in the inner ear on preFLAIR (Fig. S2), (2-2) presence and degree of enhancement in the inner ear on postFLAIR (Fig. S3), and (2-3) presence of enhancement in the inner ear on postT1WI (Fig. S4a); (3) presence of enhancement of cranial nerves or dura on either postFLAIR or postT1WI (Fig. S4b,c); and, (4) presence of enhancement of the nasopharynx, external auditory canal, and/or regional soft-tissue on postT1WI (Fig. S4d). Effusion was defined as soft-tissue filling the mastoid and middle ear cavity with T2-high and T1-low signal intensities. To assess the amount of effusion, the degree of opacification in the mastoid and middle ear was determined using four grades of increasing severity: none, mild, moderate, and severe. The degree of inner ear postFLAIR-enhancement was scored according to four grades of increasing severity based on the work of Saat et al.: none, minimal, mild, and intense^22^. Ears were bilaterally evaluated in each patient. MRI features were considered positive if present on either side.

We observed the presence of prominent nodular enhancement close to the external part of the basal turn of the cochlea at the round window (RW) level on postFLAIR and postT1WI (Fig. S5). Based on a recent study by Dubrulle et al.^23^, we conjectured that this observation was equivalent to the “RW sign”, which is a hypersignal area encompassing the RW on delayed contrast-enhanced FLAIR. In addition, several patients presented with a linear hypersignal along the vestibular aqueduct (VA) on postFLAIR (Fig. S6). The presence of the RW sign on postFLAIR and postT1WI, and the presence of VA enhancement on postFLAIR were evaluated by the two radiologists. Final decisions were reached by consensus.

### Statistical analyses

Categorical MRI findings were compared among groups using the Chi-square test and linear-by-linear association. Interobserver agreement on the MRI findings between two radiologists was calculated using Cohen's kappa (κ) index as follows: poor agreement (κ ≤ 0.2), fair (0.2 < κ ≤ 0.4), moderate (0.4 < κ ≤ 0.6), good (0.6 < κ ≤ 0.8), and excellent (0.8 < κ ≤ 1.0)^24,25^. CART analysis was conducted to evaluate MRI features that were associated with superior differential diagnosis between AIED/AID and COM^26^. CART constructs a decision tree by splitting data repeatedly into two child nodes^9,27^. This tends to maximize class purity and homogeneity such that each child node is further classified into more homogeneous groups by selecting the variable that best splits the subgroup according to the algorithm^27^. We constructed two CART models in this study. In the first CART model, all evaluated MRI features were included. Variables related to inner ear abnormalities (i.e., contrast-enhancement on postT1WI and postFLAIR), presence of the RW sign, and postFLAIR-enhancement of VA were selected in the second model to investigate MRI-based diagnostic performance. The diagnostic performance of the decision trees using the CART model was analyzed, and sensitivity, specificity, positive predictive value, and negative predictive value of the trees were calculated. CART analyses were performed with “rpart” of R statistical software (version 3.5.3., R Project for Statistical Computing, Vienna, Austria). Other statistical analyses were performed using MedCalc software (version 11.0). Statistical significance was set at *P* < 0.05.

## Results

### Clinical characteristics

In total, 67 patients were included in this study, comprising of 18 patients in the AIED/AID group (8 males and 10 females, median age = 63 years, age range = 7–81 years), 24 patients in the COM group (11 males and 13 females, median age = 61.5 years, age range = 15–86 years), and 25 patients in the control group (6 males and 19 females; median age = 61.5 years; age range = 20–76 years). Of patients in the AIED/AID group, 11 were diagnosed with GPA, 4 with CINCA syndrome/DFNA34, and 3 with Cogan’s syndrome. All patients with GPA exhibited positive ANCA in serologic tests, and pathological confirmation was obtained from six of them via kidney and/or lung lesion biopsy (n = 3), nasopharyngeal lesion biopsy (n = 2), and nasal cavity lesion biopsy (n = 1). Of the four patients clinically diagnosed with CINCA syndrome/DFNA34, three were confirmed to have an *NLRP3* variant based on genetic testing (Table S2).

**Figure 1:**
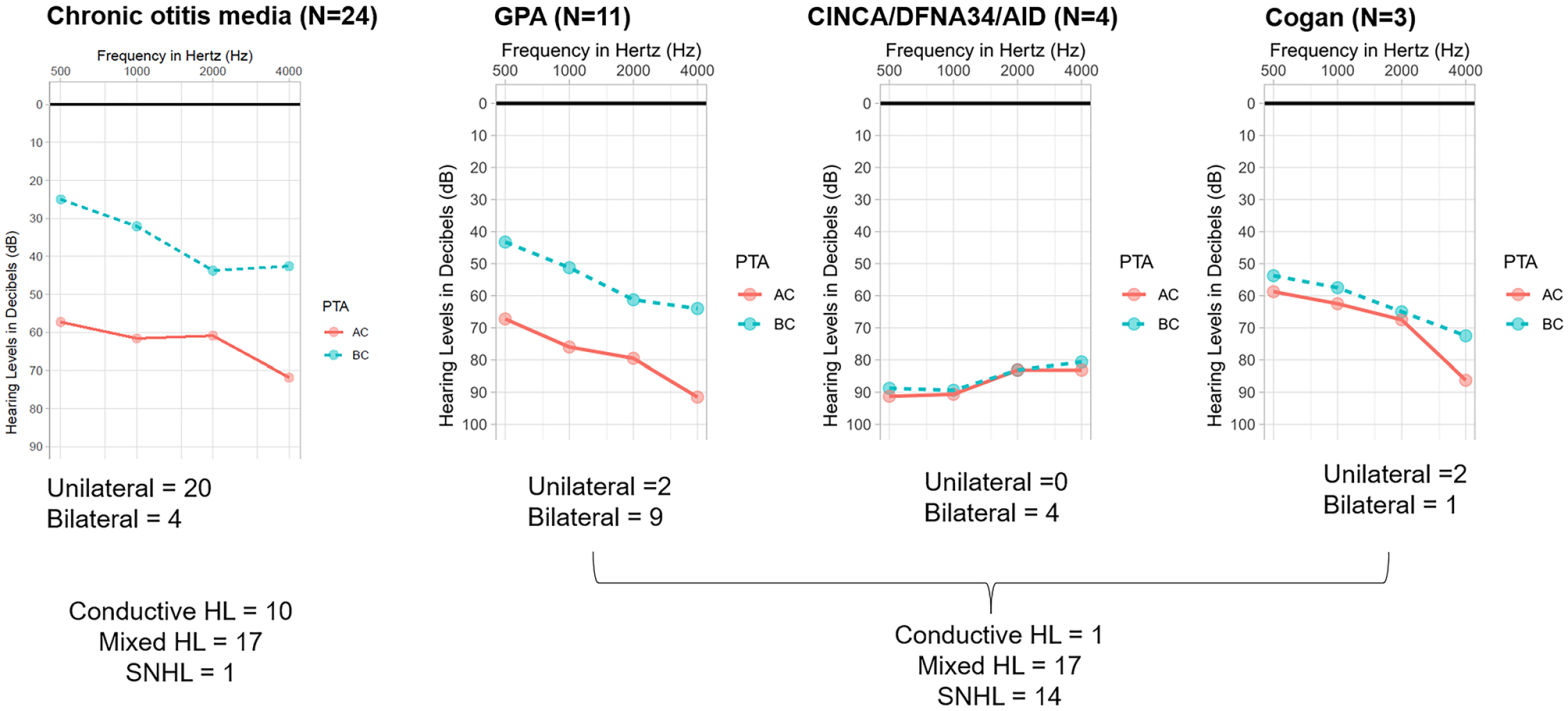
Audiogram profiles of our cohort. Audiogram profiles from subjects with chronic otitis media were not much different from those from subjects with granulomatosis polyangiitis (GPA), while CINCA/DFNA34 and Cogan syndromes manifested more of the sensorineural hearing loss (SNHL)-type profile.

In terms of audiological profiles (Fig. 1), the audiogram profile of GPA were not much different from that of COM, calling for radiological study for differential diagnosis. On the other hand, CINCA/DFNA34 and Cogan syndromes manifested more of the SNHL-type audiogram profile.

### MRI findings according to clinical diagnosis

All patients underwent heavily T2WI, pre-contrast T1WI, and postT1WI. PostFLAIR were obtained in 22 of 24 patients with COM, 16 of 18 patients with AIED/AID, and 12 of 25 control participants. PreFLAIR were unavailable in 18 of 24 patients with COM, 11 of 18 patients with AIED/AID, and 22 of 25 control participants.

Interobserver agreement was excellent for all MRI features (κ > 0.8). Consensus MRI features according to the clinical diagnosis are summarized in Table 1 and Table S3. Effusion in the mastoid and middle ear was present in all patients in the COM group (100%) and in 72.2% of patients in the AIED/AID group. Effusion was absent in most control participants (92.0%). The presence of effusion was predominantly unilateral in the COM group (87.5%) and predominantly bilateral in the AIED/AID group (61.1%). The amount of effusion was larger in the COM group than in the AIED/AID group (100% in 16 and 5 patients, respectively).

**Table 1:** MRI findings according to the clinical diagnosis.

	COM group (n = 24)	AIED/AID group (n = 18)	Control group (n = 25)	*P*-value
**Laterality of mastoid and middle ear effusion**				
Absent	0 (0)	5 (27.8)	23 (92.0)	< 0.0001*
Unilateral	21 (87.5)	2 (11.1)	1 (4.0)	
Bilateral	3 (12.5)	11 (61.1)	1 (4.0)	
**Amount of mastoid and middle ear effusion**				
None	0 (0)	5 (27.8)	25 (100)	< 0.0001*
Mild	3 (12.5)	3 (16.7)	0 (0)	
Moderate	5 (20.8)	5 (27.8)	0 (0)	
Severe	16 (66.7)	5 (27.8)	0 (0)	
**Presence of any abnormalities in the inner ear**				
Absent	18 (75.0)	0 (0)	24 (96.0)	< 0.0001*
Present	6 (25.0)	18 (100)	1 (4.0)	
**Location of inner ear pathology**				
Absent	18 (75.0)	0 (0)	24 (96.0)	< 0.0001*
Cochlea alone	4 (16.7)	4 (22.2)	1 (4.0)	
More than two subsites	2 (8.3)	14 (77.8)	0 (0)	
**Laterality of inner ear pathology**				
Absent	18 (75.0)	0 (0)	24 (96.0)	< 0.0001*
Unilateral	2 (8.3)	1 (5.5)	0 (0)	
Bilateral	4 (16.7)	17 (94.5)	1 (4.0)	
**Presence of preFLAIR inner ear hyperintensity**				
Absent	4 (16.7)	0 (0)	3 (12.0)	0.005*
Present	2 (8.3)	7 (38.9)	0 (0)	
Not available	18 (75.0)	11 (61.1)	22 (88.0)	
**Presence of postFLAIR inner ear enhancement**				
Absent	16 (66.7)	0 (0)	11 (44.0)	< 0.0001*
Present	6 (25.0)	16 (88.9)	1 (4.0)	
Not available	2 (8.3)	2 (11.1)	13 (52.0)	
**Degree of postFLAIR inner ear enhancement**				
None	16 (66.7)	0 (0)	11 (44.0)	< 0.0001*
Minimal	5 (20.8)	2 (11.1)	1 (4.0)	
Mild	1 (4.2)	2 (11.1)	0 (0)	
Intense	0 (0)	12 (66.7)	0 (0)	
Not available	2 (8.3)	2 (11.1)	13 (52.0)	
**Presence of postT1WI inner ear enhancement**				
Absent	24 (100)	7 (38.9)	25 (100)	< 0.0001*
Present	0 (0)	11 (61.1)	0 (0)	
**Presence of cranial nerve involvement on postT1WI**				
Absent	24 (100)	15 (83.3)	25 (100)	0.0139*
Present	0 (0)	3 (16.7)	0 (0)	
**Presence of dura involvement on postT1WI**				
Absent	23 (95.8)	12 (66.7)	25 (100)	0.0009*
Present	1 (4.2)	6 (33.3)	0 (0)	
**Presence of nasopharynx, external auditory canal, and regional soft-tissue involvement on postT1WI**				
Absent	23 (95.8)	9 (50)	25 (100)	< 0.0001*
Present	1 (4.2)	9 (50)	0 (0)	
**Presence of RW sign on postFLAIR**				
Absent	17 (70.9)	5 (27.8)	12 (48)	0.0001*
Present	5 (20.8)	11 (61.1)	0 (0)	
Not available	2 (8.3)	2 (11.1)	13 (52.0)	
**Presence of RW sign on postT1WI**				
Absent	22 (91.7)	8 (44.4)	25 (100)	< 0.0001*
Present	2 (8.3)	10 (55.6)	0 (0)	
**Presence of VA enhancement on postFLAIR**				
Absent	20 (83.4)	7 (38.9)	12 (48)	0.0001*
Present	2 (8.3)	9 (50.0)	0 (0)	
Not available	2 (8.3)	2 (11.1)	13 (52.0)	

Data are presented as n (%).

*MRI* magnetic resonance imaging, *COM* chronic otitis media, *AIED/AID* autoimmune/autoinflammatory inner ear disease, *preFLAIR* pre-contrast fluid-attenuated inversion recovery image, *postFLAIR* post-contrast fluid-attenuated inversion recovery image, *postT1WI* post-contrast T1-weighted image, *RW sign* round window sign, *VA* vestibular aqueduct.

*Statistical significance was set at *P* < 0.05.

All patients in the AIED/AID group (100%) presented with abnormal findings on either side of inner ear structures. Division of inner ear structures into the cochlea, vestibule, and semicircular canal revealed that 77.8% of the AIED/AID group presented with involvement of more than two subsites, and 94.5% presented with bilateral involvement of inner ear structures. The prevalence of preFLAIR hyperintensity and postFLAIR-enhancement was significantly higher in the AIED/AID group than in the COM group (when available, 100% and 93.8% versus 33.3% and 27.3%, respectively). The degree of postFLAIR-enhancement was minimal to mild in all patients with COM and was significantly greater (intensive in 66.8%) in patients with AIED/AID. The presence of postT1WI-enhancement in the inner ear was observed exclusively in the AIED/AID group (61.1%). No inner ear postT1WI-enhancement was observed in the COM and control groups.

An ancillary but notable finding was the presence of cranial nerve enhancement on postT1WI in 3 of 18 patients in the AIED/AID group. No such observations were noted in the COM or control groups. The frequency of cranial nerve enhancement was significantly higher in the AIED/AID group than in the COM and control groups (*P* = 0.014). Abnormal dural postT1WI-enhancement was significantly greater in the AIED/AID group (33.3%) than in the COM (4.2%) and control groups (0%). Abnormal cranial nerve enhancement was observed in 2 of 4 patients (50.0%) with CINCA syndrome/DFNA34 and 1 of 11 patients (9.1%) with GPA. The frequency of dural enhancement was significantly higher in patients with GPA (45.5%) than Cogan’s syndrome or CINCA syndrome/DFNA34. One of three patients with Cogan’s syndrome and one patient in the COM group presented with abnormal dural enhancement. The prevalence of abnormal enhancement in the adjacent nasopharynx, external auditory canal, and regional soft-tissue area was significantly higher in the AIED/AID group (50%), especially in patients with GPA (7 of 11 patients) than other two subsets.

The frequency of the RW sign in postFLAIR and postT1WI was significantly higher in the AIED/AID group than in the COM group. None of the control participants presented RW sign. Among the patients with available postFLAIR, VA enhancement was present in 9 of 16 patients with AIED/AID; this prevalence was significantly higher than that in the COM group (2 of 22 patients).

### MRI-based decision tree

**Figure 2:**
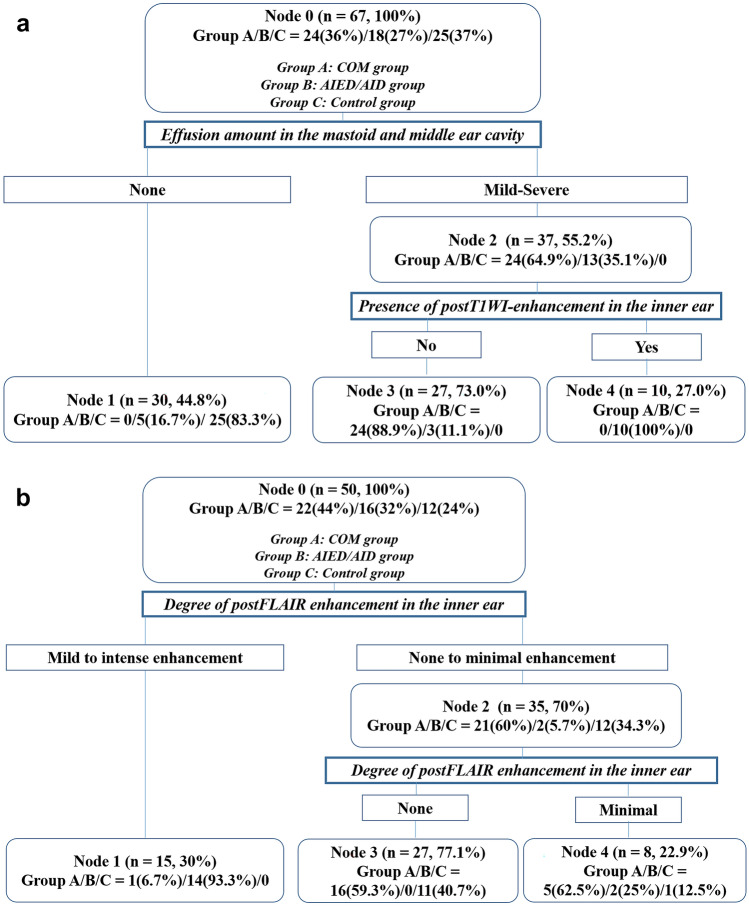
Classification and regression tree analysis (CART) results. The first decision-making tree (**a**) uses two nodes of the amount of effusion in the mastoid and middle ear cavity and the presence of enhancement in the inner ear structures on post-contrast T1-weighted images (postT1WI). The second decision-making tree (**b**) utilized the degree of enhancement in the inner ear structures on post-contrast FLAIR images (postFLAIR) as the partitioning node. Group A was the chronic otitis media (COM) group, group B was the autoimmune inner ear disease and autoinflammatory inner ear disease (AIED/AID) group, and group C was the control group. The details regarding the tree are explained in the “Results” section.

We constructed a first model using all MRI features (summarized in Table 1). In this decision tree model (Fig. 2a), the amount of effusion in the mastoid and middle ear cavities was used as the first partitioning node. Of the 67 patients, 37 (55.2%) presented with mild to severe degree of the effusion. For these patients, the next branching node was the presence of inner ear enhancement on postT1WI. Of these 37 patients, 27 (73.0%) did not present postT1WI-enhancement in the inner ear; 24 of these 27 patients (88.9%) were in the COM group and three (11.1%) were in the AID/AIED group. Of the 37 patients in the second node, 10 (27.0%) who presented with inner ear enhancement on postT1WI were all in the AID/AIED group (100%). The sensitivity, specificity, positive predictive value, and negative predictive value for each clinical diagnosis in the first tree model are presented in Table 2.

**Table 2:** Diagnostic performance of the two decision-making trees.

	SN (%) (95% CI)	SP (%) (95% CI)	PPV (%) (95% CI)	NPV (%) (95% CI)
**The first decision-making tree**^a^				
(COM group) vs. (AIED/AID, control groups)	100 (0–100)	93 (38.8–85.4)	88.9 (60.5–77.0)	100 (0–100)
(AIED/AID group) vs. (COM, control groups)	55.6 (32.6–78.5)	100 (0–100)	100 (0–100)	86 (77–95)
(Control group) vs. (COM, AIED/AID groups)	100 (0–100)	88.1 (78.3–97.9)	88.3 (70–97)	100 (0–100)
**The second decision-making tree**^b^				
(COM group) vs. (AIED/AID, control groups)	22.7 (52.1–40.2)	91.3 (80.0–100)	71.4 (38.0–100)	55.3 (39.4–71.1)
(AIED/AID group) vs. (COM, control groups)	40 (0–83.0)	94.5 (92.7–100)	66.7 (13.3–100)	92.8 (85.1–100)
(Control group) vs. (COM, AIED/AID groups)	94.4 (83.9–100)	33.3 (15.5–51.1)	48.6 (32.0–65.1)	90 (71.4–100)

*SN* sensitivity, *SP* specificity, *PPV* positive predictive value, *NPV* negative predictive value, *CI* confidence interval, *COM* chronic otitis media, *AIED/AID* autoimmune/autoinflammatory inner ear disease.

^a^The first decision-making tree used two nodes for the amount of effusion and presence of inner ear enhancement on post-contrast T1-weighted image.

^b^The second decision-making tree used two nodes of the degree of inner ear enhancement on post-contrast fluid-attenuated inversion recovery image.

The second tree model was constructed using variables related to inner ear enhancement, RW sign, and VA enhancement. In this second tree model (Fig. 2b), only the degree of postFLAIR-enhancement in the inner ear was maintained as both the first and second partitioning nodes. Among total 50 study subjects who had available postFLAIR, the degree of postFLAIR-enhancement in the inner ear was mild or intense in 15 patients (30%), the majority of which belonged to the AIED/AID group (14 of 15 patients, 93.3%). In contrast, 35 of 50 patients (70%) with absent or minimal degree of postFLAIR inner ear enhancement belonged to the COM or control groups. The sensitivity, specificity, positive predictive value, and negative predictive value for each clinical diagnosis in the second tree model are summarized in Table 2.

### Representative cases of CINCA syndrome/DFNA34/AID

Here, we present three cases representing each of the three categories of autoinflammatory inner ear disorder (CINCA syndrome/DFNA34/AID) which exhibited distinct MRI features according to the genotypes of *NLRP3* variants (Table S2). CINCA syndrome is a rare AIED with autosomal dominant inheritance, and is linked with *NLRP3* variants which cause hyper-activation of inflammasomes and excessive release of interleukin-1^3^. Monocytes, which release interleukin-1, are distributed throughout the cochlear nerve and cochlear structure; hence, *NLPR3* gene variants affect the cochlear nerve depending on the degree of pathogenic potential.

#### Case 1

**Figure 3:**
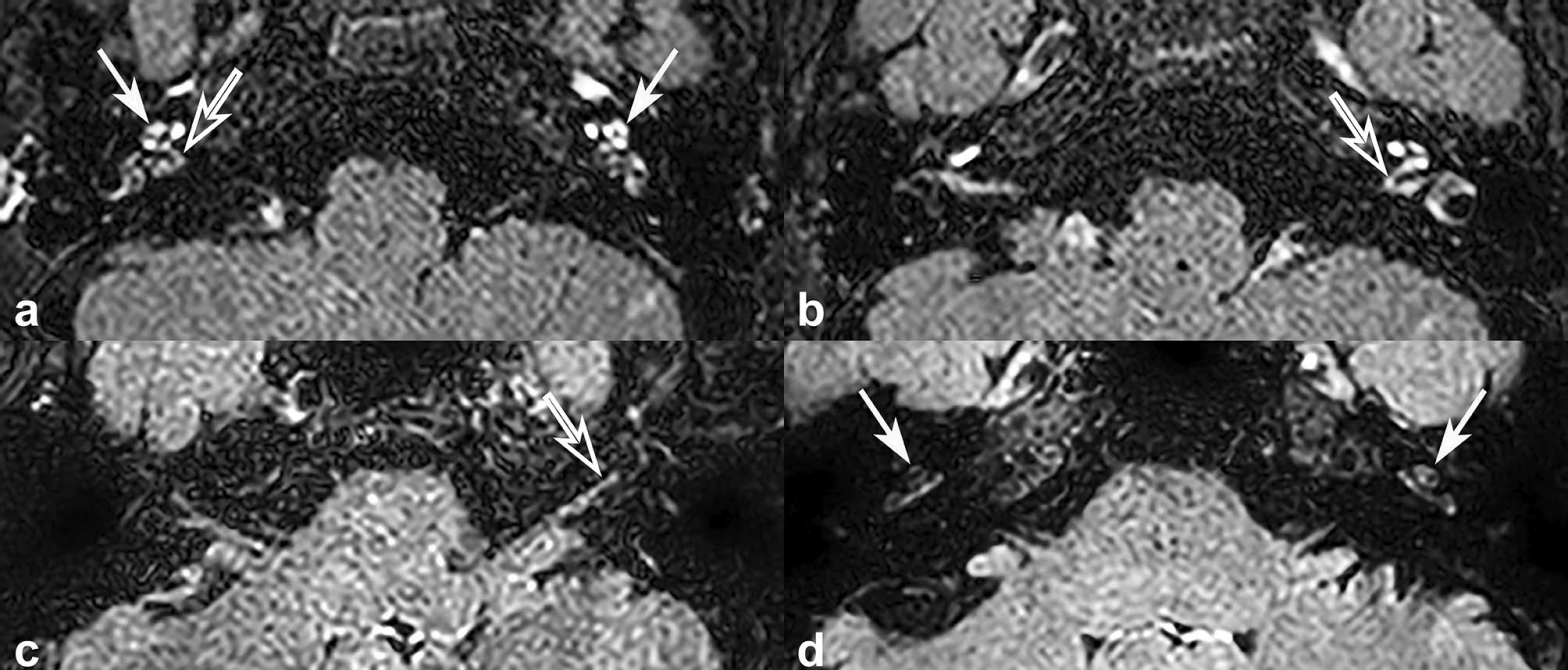
Representative cases of CINCA syndrome. (**a**,**b**) A 13-year-old male diagnosed with CINCA syndrome presented with intense inner ear enhancement in bilateral cochlea (arrows) and strong enhancement of bilateral cochlear nerves (empty arrows) on post-contrast FLAIR image (postFLAIR). (**c**) A 42-year-old female diagnosed with CINCA syndrome presented with strong enhancement of left cochlear nerve on postFLAIR (empty arrow) compared to enhancement of the cochlea. (**d**) A 38-year-old female diagnosed with CINCA syndrome presented with postFLAIR-enhancement of bilateral cochlea (arrows), but absence of enhancement of the cochlear nerve.

A 13-year-old male was diagnosed with full-blown CINCA due to a de novo variant of *NLRP3* with high expressivity. Intense postFLAIR-enhancement was observed in the cochlea (arrows), vestibule, and semicircular canal, and strong enhancement involving the cochlear nerve (empty arrows) was also noted (Fig. 3a,b).

#### Case 2

A 42-year-old female was diagnosed with DFNA34, a non-syndromic form of hearing loss due to an *NLRP3* variant residing in the LPR domain possibly with lower pathogenic potential than CINCA syndrome. Although not detectable on postT1WI, postFLAIR revealed stronger enhancement in the cochlear nerve than in the cochlea (Fig. 3c).

#### Case 3

A 38-year-old female was strongly suspected of having AID clinically. Although we defined some evidence that suggested mosaicism of a *NLRP3* variant from this case, the presence of mosaicism was not confirmed yet, making this case 3 to be the one without detectable *NLRP3* variant at this point. Minimal postFLAIR-enhancement was observed in the cochlea, but no demonstrable enhancement of the cochlear nerve was noted (Fig. 3d).

## Discussion

This study is the first to evaluate MRI findings in patients with AIED/AID and concomitant SNHL, including patients with GPA, CINCA syndrome/DFNA34, and Cogan’s syndrome. We observed that the frequency of bilateral postFLAIR- and postT1WI-inner ear enhancements, RW sign, and VA enhancement was higher in patients with AIED/AID than in patients with COM and control participants. Further, the frequency of regional soft-tissue enhancement was significantly higher in patients with GPA than in other patients with AIED/AID. Our CART decision tree model demonstrated that the amount of bilateral effusion in the mastoid and middle ear cavity and the presence and degree of inner ear enhancement on postFLAIR and postT1WI enabled differentiation of AIED/AID from COM accompanied by SNHL with high specificity. Our findings support the employment of this simple tree model to facilitate radiologic decision-making for patients with AIED/AID and COM. Especially, GPA comprised more than half of the total AIED/AID cases. The similar audiogram profile of GPA with that of COM raises the unmet clinical needs to differentiate one from the other, highlighting the importance of this simple tree model.

Sensory organs in the inner ear can be involved by autoimmune or autoinflammatory processes^28,29^. The main pathogenesis of AIED/AID is thought to be self-aggregation of T lymphocytes against specific antigens of the inner ear, forming circulating autoantibodies^28,29^. Nonetheless, revealing autoimmunity in AIED/AID is challenging, since there are few tests that can detect specific autoantibodies. Therefore, current diagnosis of AIED/AID is based either on clinical criteria or prompt response to the steroid therapy. Meanwhile, autoimmunity has also been proposed as one of the etiologies of Meniere’s disease—a clinical syndrome defined with endolymphatic hydrops and characterized by symptoms of vertigo, tinnitus, and SNHL^28,29^. Despite the strong evidence that immune mechanisms are involved in Meniere’s disease, its exact pathogenetic mechanism is still unclear, and many other etiologies such as viral infection are considered as the cause of the disease^28,29^. Accordingly, there are many treatment options for Meniere’s disease including diuretics, diazepam, corticosteroid, immunosuppressive agent, and even labyrinthectomy. Therefore, to avoid possible overlap with AIED/AID, we decided to completely exclude patients with the diagnosis of probable/possible Meniere’s disease from our study. In this regard, we also excluded patients who solely presented vestibular symptoms without auditory symptoms.

Unlike Meniere’s disease the diagnosis of which can be supported by demonstrating endolymphatic hydrops on delayed contrast-enhanced MRI^30^, there is a paucity of radiologic research on AIED/AID, with the exception of several case reports. Teszler et al. reported a case of GPA with bilateral otitis media and rapidly progressive SNHL; the patient presented with postT1WI-enhancement in bilateral cochlear basal turns, which subsequently resolved with immunosuppressive therapy^31^. Yildirim et al. reported a case of GPA presenting with bilateral cochlear and leptomeningeal enhancement, which was in accordance with our findings^32^. Radiologic findings of Cogan’s syndrome have been reported by Casselman et al. including obliteration of the intralabyrinthine fluid spaces with calcification formation and high signal intensity and enhancement of the cochlea and vestibule on pre- and post-contrast T1WI^33^.

Nevertheless, reports of relevant MRI findings are scarce and fail to distinguish AIED/AID from other etiologies such as labyrinthitis accompanied by COM. In patients with COM, dysfunction in sound transmission from the middle ear to inner ear results in conductive hearing loss. Patients with COM may also develop SNHL due to cochlear damage or altered sound wave transmission mechanisms in the ear^34^. Thus, for cases of SNHL with COM in the absence of clinical signs indicating COM such as tympanic membrane perforation, distinguishing inner ear involvement due to AIED/AID from that due to COM is challenging.

In this context, we hypothesized that specific MRI findings could be used to differentiate between AIED/AID and COM. We therefore generated MRI-based decision trees to differentiate these two conditions. In our first tree model, the amount of effusion in the mastoid and middle ear cavities was used as the first partitioning node. Radiologic reports of mastoid or middle ear cavity effusion in patients with AIED/AID are lacking, but it is known that the most frequent manifestations of COM are mastoid and middle ear cavity effusion^35,36^. Accordingly, our study demonstrated that the proportion of patients with effusion amount more than moderate degree was higher in the COM group than in the AIED/AID group. Conversely, approximately half of the AIED/AID group presented with effusion more than moderate degree, and when present, bilateral effusion was more frequent. The pathogenesis of COM involves dysfunction of ventilation of the middle ear cavity and mastoid air cells due to Eustachian tube impairment. Therefore, COM commonly manifests as a large amount of unilateral effusion and persistent inflammation of the middle ear or mastoid cavity^37,38^. AIED/AID are underscored by pathology of the inner ear but not middle ear; hence, the amount of effusion is smaller but frequency of bilateral effusion is higher than that in COM given the systemic nature of AIED/AID.

The second partition of the first tree model was postT1WI-enhancement in inner ear structures. The presence of postT1WI-enhancement predicted AIED/AID with high diagnostic performance. Indeed, cochlear enhancement has been reported in previous case studies of AIED^39–43^. This result was strongly associated with the partitioning nodes in the second tree model, which constituted postFLAIR-enhancement of inner ear structures; here, most patients in the AIED/AID group exhibited postFLAIR-enhancement with high intensity. In contrast, the frequency of postFLAIR-enhancement was lower in the COM group than in the AIED/AID group. These results could be due to the underlying pathogenesis of these diseases. It has been proposed that uncontrolled attack on inner ear proteins results in proinflammatory T-cell responses and autoantibody formation in AIED/AID^3^. In addition, lymphocytes activated by autoimmune responses cross the blood–labyrinthine barrier and reach the endolymphatic sac^44^. Accordingly, AIED/AID are frequently accompanied by SNHL at early stages, leading to active inflammation of the inner ear. Indeed, the occurrence of inner ear enhancement was more frequent and its degree was more severe in patients with AIED/AID than in patients with COM. In particular, postFLAIR is more sensitive to inner ear enhancement from the impairment of the blood–labyrinthine barrier^45^, given that postFLAIR is more sensitive to low concentrations of gadolinium-contrast compared to postT1WI. In this regard, we observed intense enhancement on postFLAIR only in the majority of patients with AIED/AID, and postT1WI-enhancement was present exclusively in the AIED/AID group.

The frequency of the RW sign on postFLAIR and/or postT1WI was significantly higher in the AIED/AID group than in the COM group. Dubrulle et al. reported the use of the RW sign as a marker for perilymphatic fistula resulting in contrast pooling in the RW region^23^. Attye et al. reported that the RW sign was observed following obliteration of the RW due to inflammation^46^. We thus hypothesized that the presence of nodular enhancement at the RW on immediate postFLAIR and postT1WI could be due to significant inflammation at the RW level. Our results suggest that inflammation in the RW as well as inner ear may be more severe in patients with AIED/AID than in patients with COM, due to increased permeability and diffusion of microtoxins into the inner ear^34^. Additionally, the frequency of VA enhancement on postFLAIR was significantly higher in the AIED/AID group. Given that the endolymphatic sac is associated with AIED pathogenesis^44^, VA enhancement on postFLAIR suggests profound inflammation of the endolymphatic sac in AIED/AID than in COM-associated labyrinthitis.

We noted differences in the prevalence of MRI findings among AIED/AID subsets (Table S2). Abnormal enhancement involving the dura, nasopharynx, external auditory canal, and regional soft-tissue area were significantly more frequent in patients with GPA. In GPA, diffuse meningeal enhancement and abnormal soft-tissue enhancement in the nasopharynx have been reported in previous studies^47–50^. In addition, the amount of effusion tended to be larger in patients with GPA than in those with Cogan’s syndrome or CINCA syndrome/DFNA34, presumably due to Eustachian tube dysfunction resulting from soft-tissue involvement by the disease. Cranial nerve enhancement was noted in one of the patients with GPA, which verifies cranial nerve involvement and agrees with reports of cranial palsy in patients with GPA^51,52^. Patients with Cogan’s syndrome exhibited intense bilateral inner ear enhancements, and one patient presented with dura and adjacent soft-tissue enhancements. Our findings are in accordance with previous reports indicating that Cogan’s syndrome manifests with meningoencephalitis, cranial neuropathy, and inner ear enhancement on MRI^39–42^. Moreover, intense inner ear enhancement was observed in patients with CINCA syndrome. Abnormal cochlear nerve enhancement was dependent on the *NLRP3* genotypes, which probably have resulted in different degree of ramifications. This was in agreement with the findings of Behringer et al., who reported multiple cranial nerve enhancement and cochlear enhancement in a patient with CINCA syndrome^43^. Consequently, we speculate that subcategories of AIED/AID can be differentiated using specific MRI findings, but further studies are required to validate these results.

Our study has several limitations. First, although we included patients diagnosed with AIED/AID from a nationwide tertiary referral hospital over a decade, the sample size was relatively small. However, the low prevalence of AIED/AID is a fundamental limiting factor in this regard. Further, the prevalence of GPA was higher than that of Cogan’s syndrome or CINCA syndrome in the AIED/AID group, also due to the low prevalence of the syndrome. Future multi-center studies with larger sample sizes are warranted. Second, we could not perform a power analysis for the calculation of the optimal sample size, since our study was not based on the testing of hypothesis but adopted a prediction model of decision-making tree in a retrospectively pooled study population. Future study will be necessary to validate reproducibility of our study results. Third, we used different MRI machines and coils with different channels, which might affect the image quality. However, all machines were based on 3 T, and despite the difference in the coils, interobserver agreement on the visual image analysis between the radiologists was excellent. Therefore, we believe that this difference had little effect on the results. Fourth, although it is well known that pro-inflammatory cytokines are involved in the pathogenesis of AIED/AID and can confirm the diagnosis, we could not obtain data on the cytokines in our study population. Future researches that integrate the measurement of cytokines and MRI findings are expected to improve the diagnostic performance of the decision tree differentiating AIED/AID. Lastly, our study focused on radiologic differentiation between inner ear involvement by AIED/AID and COM. As such, we did not evaluate the relationship between MRI findings and clinical symptom severity or prognosis post-treatment. Future studies are warranted to verify clinico-radiological associations in AIED/AID.

## Conclusions

The amount of effusion, postT1WI- and postFLAIR-inner ear enhancement, RW sign, and VA enhancement on postFLAIR may differentiate AIED/AID (including GPA, Cogan’s syndrome, and CINCA syndrome/DFNA34) from COM presenting with SNHL. Our high-resolution MRI-based CART model may be a useful tool for diagnosing and treating patients with AIED/AID and COM that present similar clinical symptoms.

## Supplementary Information

Supplementary Figure S1.

Supplementary Figure S2.

Supplementary Figure S3.

Supplementary Figure S4.

Supplementary Figure S5.

Supplementary Figure S6.

Supplementary Information.

## Data Availability

Data are only available upon request, and before the request, data cannot be shared publicly by the regulation of Institutional Review Board of Seoul National University Bundang Hospital, because data may contain potentially identifying or sensitive patient information. For researchers who may wish to have access to data of this study, please contact via the following e-mail and send data inquiry: msri2@snubh.org (Research Support, Institutional Review Board of Seoul National University Bundang Hospital).
