# Supplementary figures and images for: Construction of an MRI-based decision tree to differentiate autoimmune and autoinflammatory inner ear disease from chronic otitis media with sensorineural hearing loss
Source: Sci Rep. 2021 Sep 27;11:19171. doi: 10.1038/s41598-021-98557-w (PMC8476614; doi:10.1038/s41598-021-98557-w)

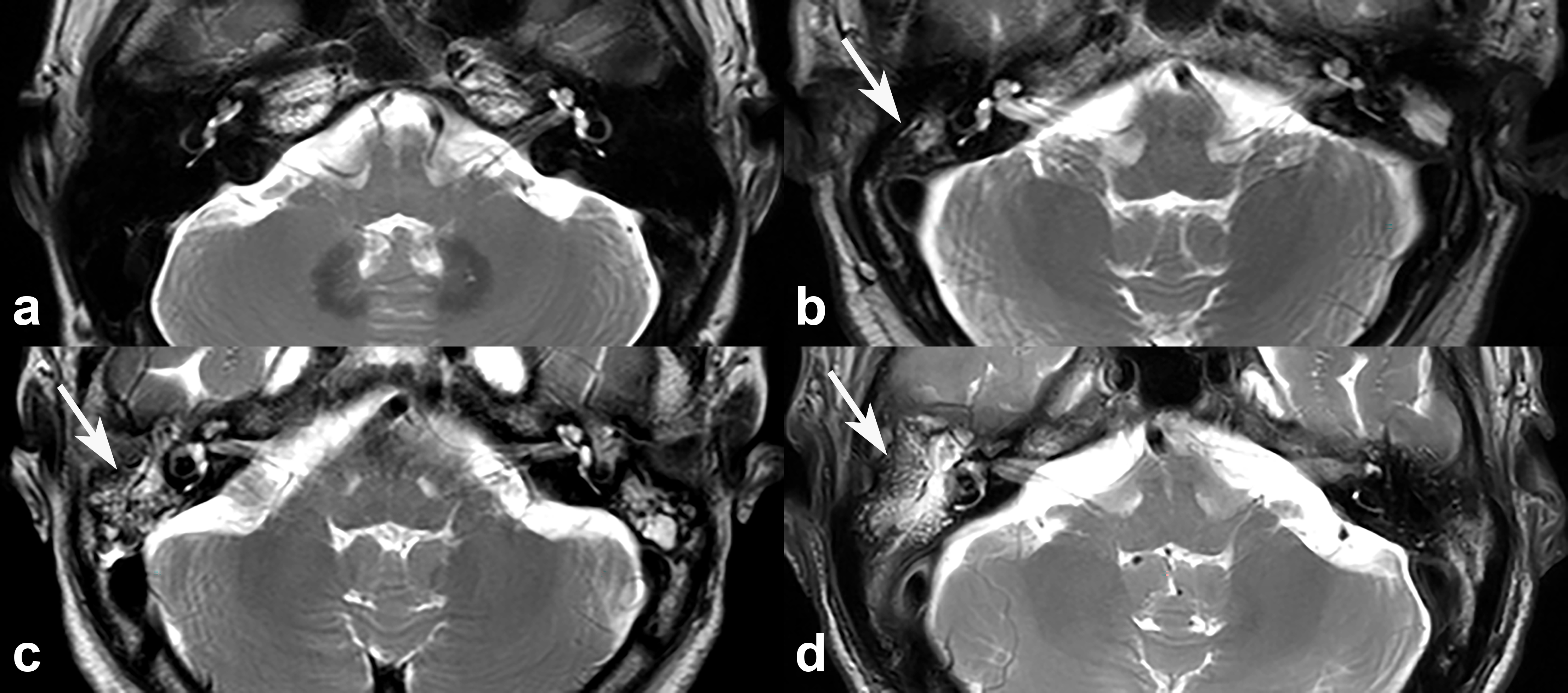

Supplement: Supplementary file 1 — Supplementary Figure S1. [file 41598_2021_98557_MOESM1_ESM.tif]

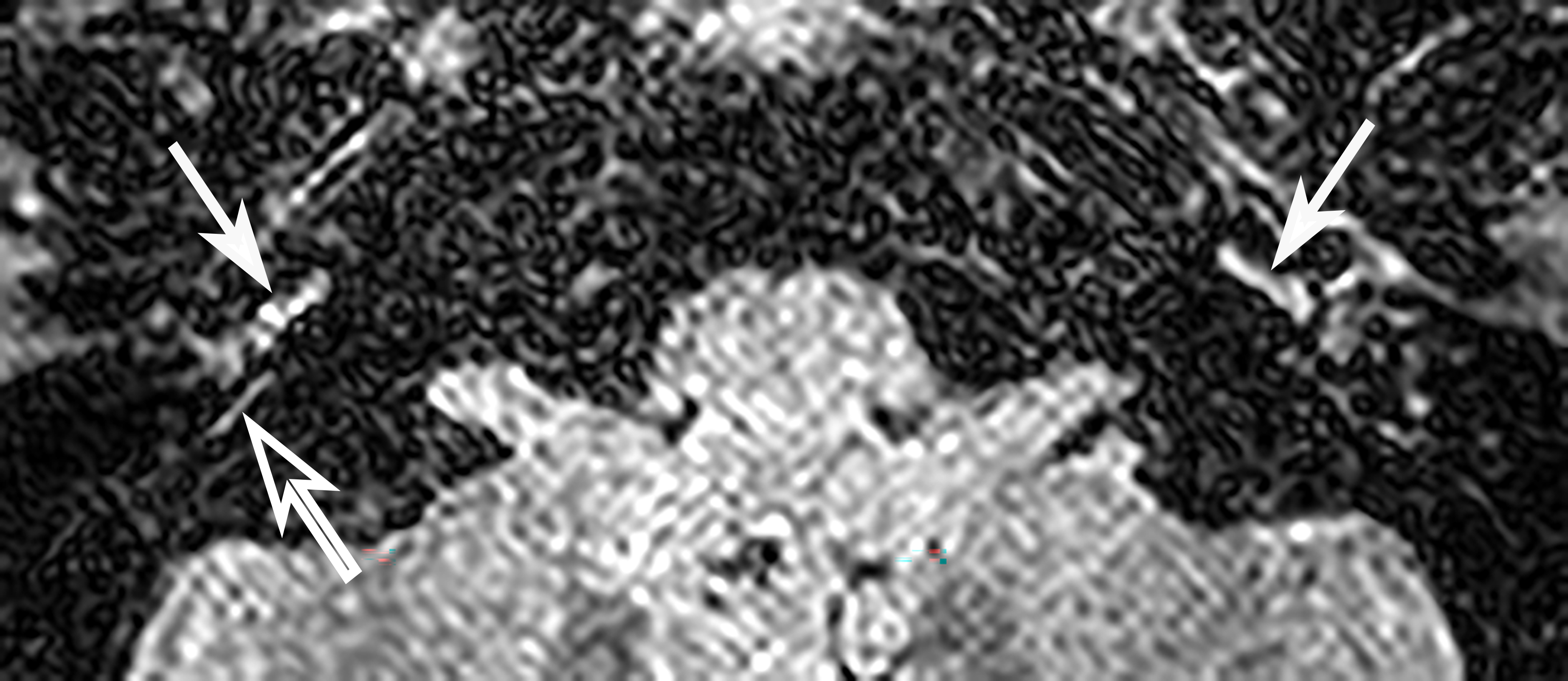

Supplement: Supplementary file 2 — Supplementary Figure S2. [file 41598_2021_98557_MOESM2_ESM.tif]

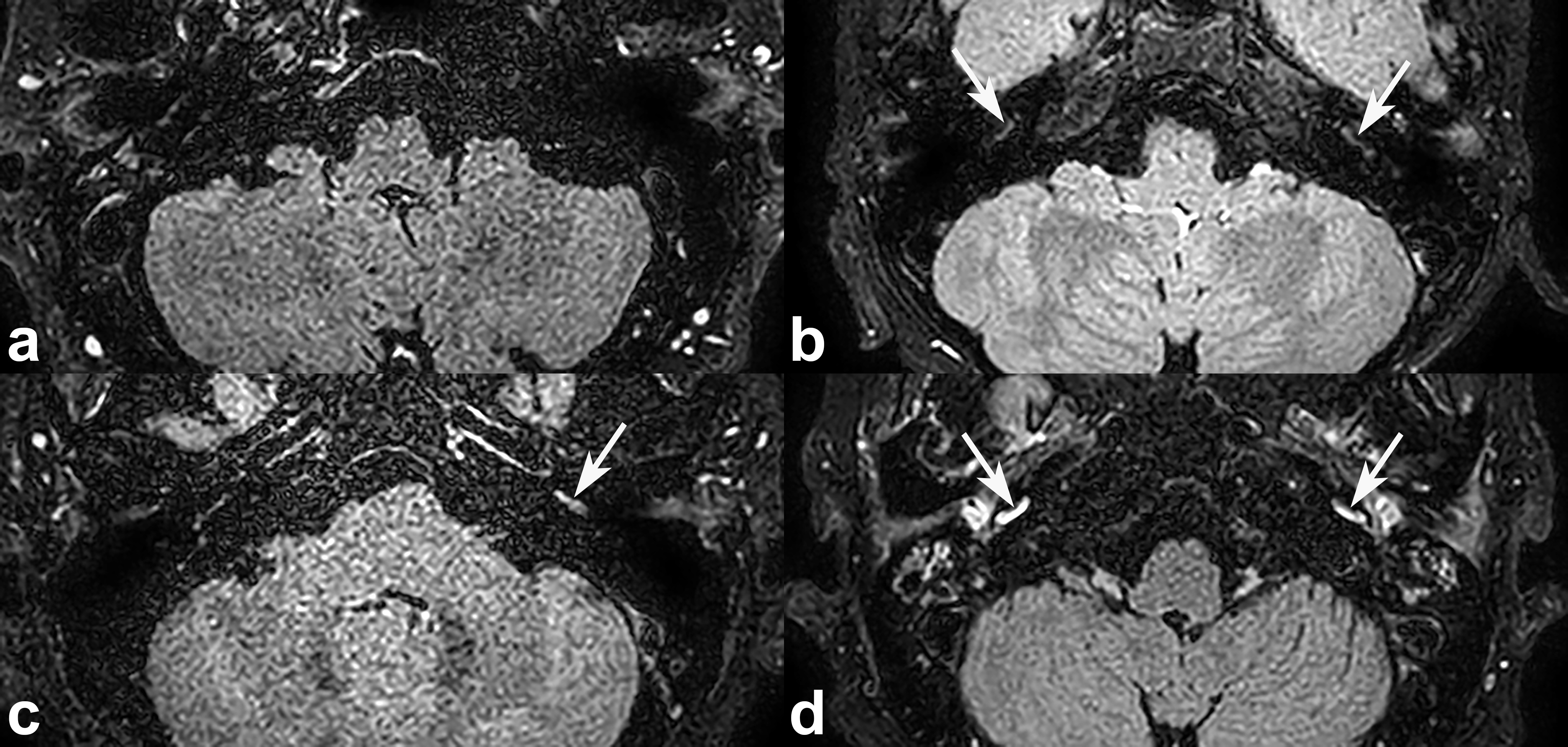

Supplement: Supplementary file 3 — Supplementary Figure S3. [file 41598_2021_98557_MOESM3_ESM.tif]

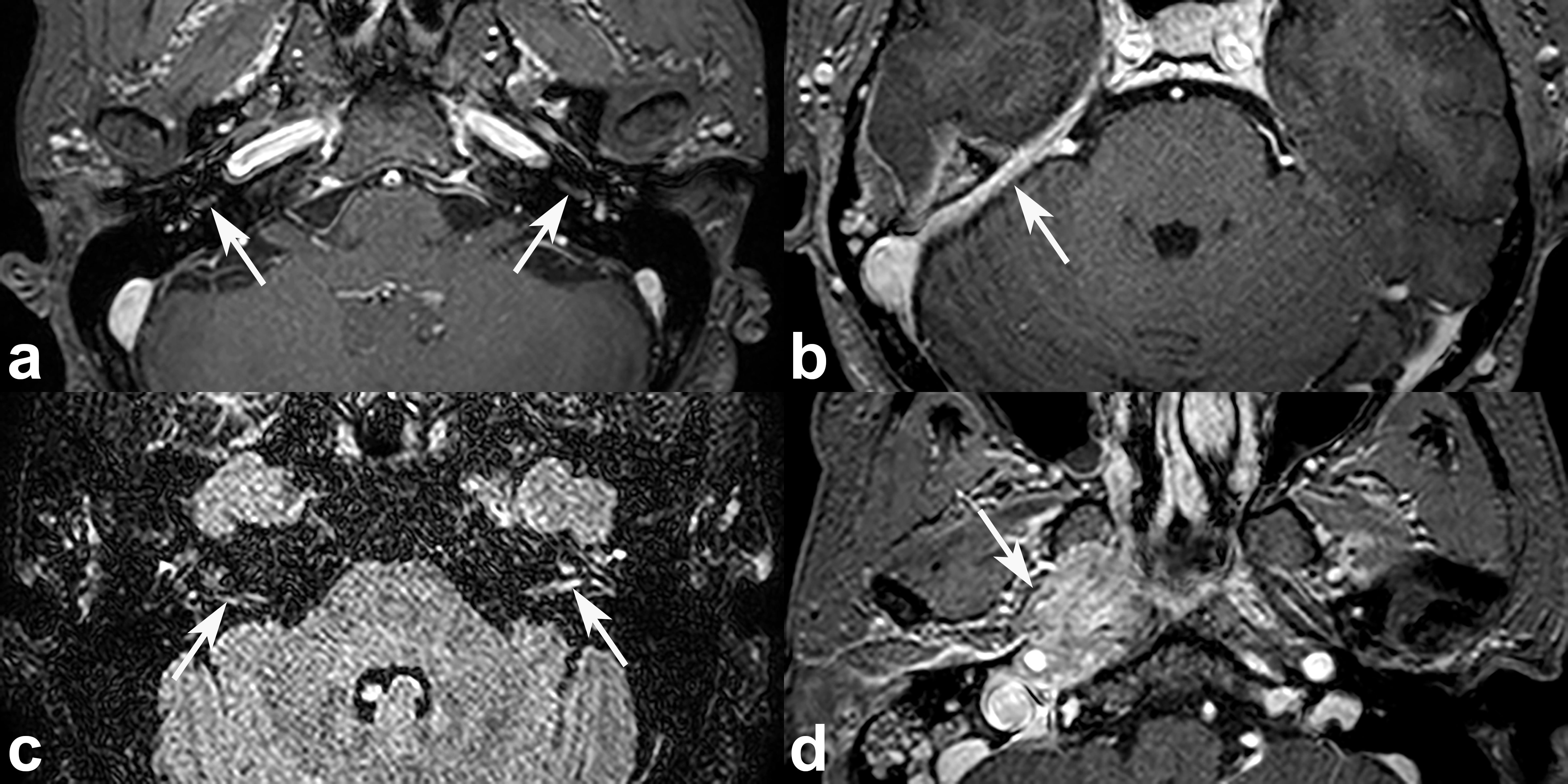

Supplement: Supplementary file 4 — Supplementary Figure S4. [file 41598_2021_98557_MOESM4_ESM.tif]

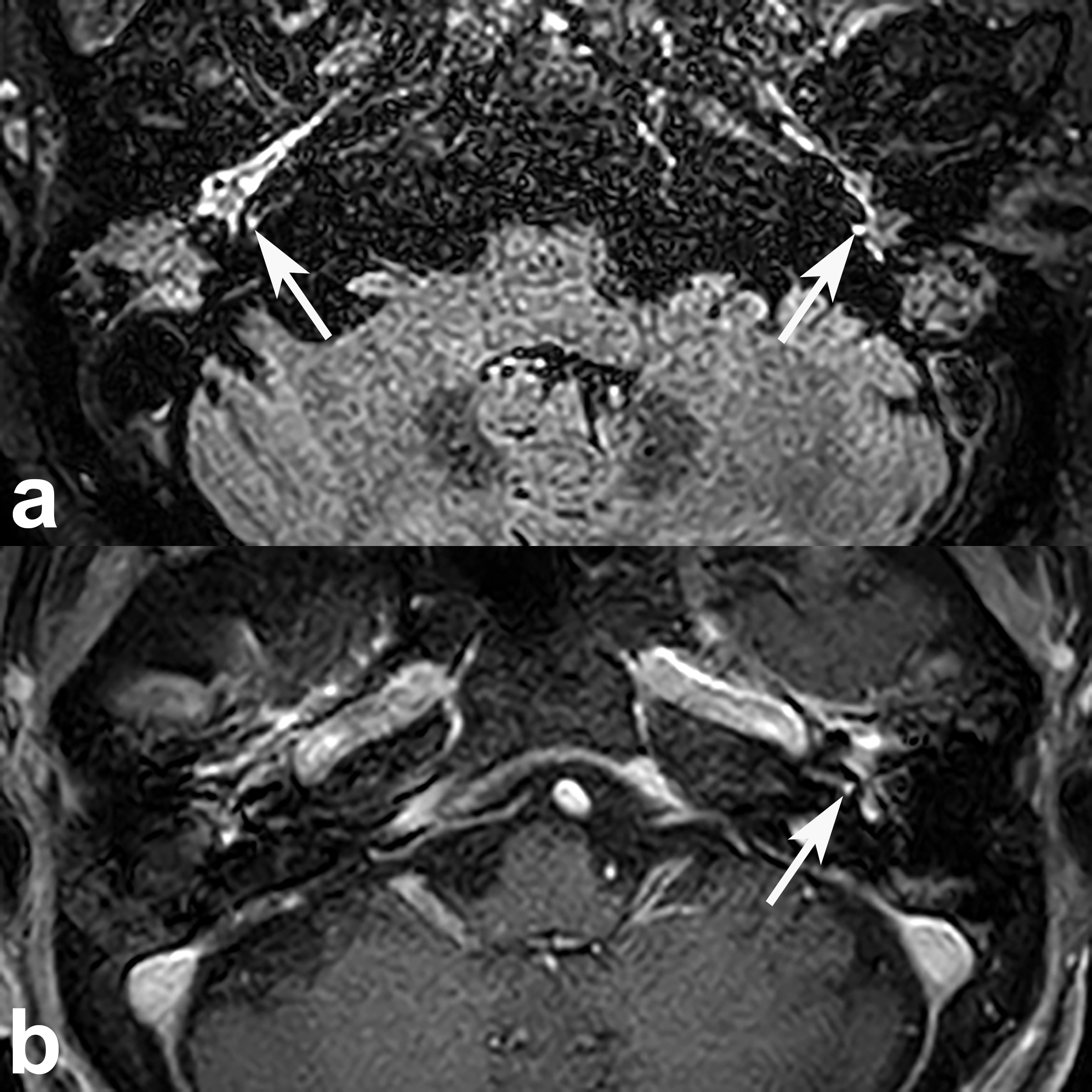

Supplement: Supplementary file 5 — Supplementary Figure S5. [file 41598_2021_98557_MOESM5_ESM.tif]

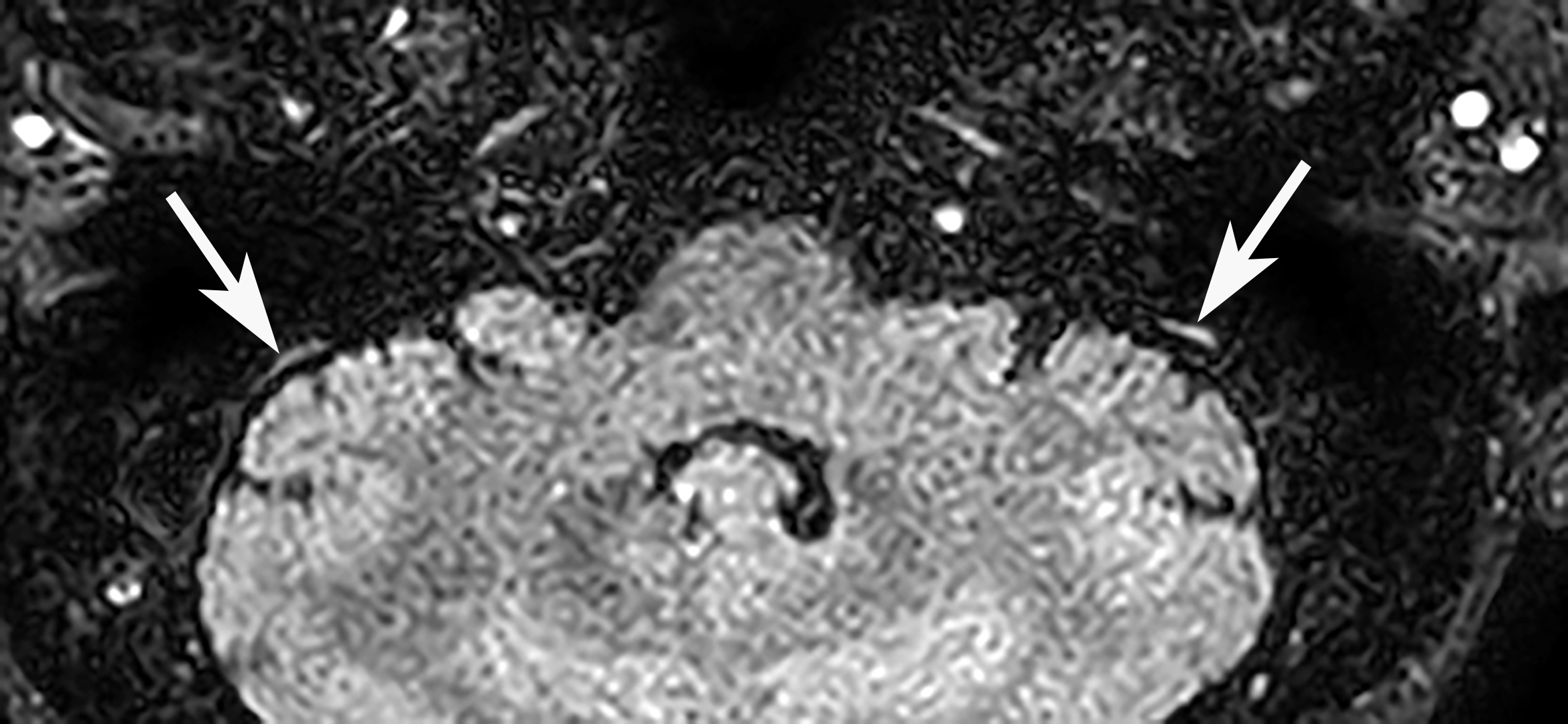

Supplement: Supplementary file 6 — Supplementary Figure S6. [file 41598_2021_98557_MOESM6_ESM.tif]
