# Supplementary material for: Construction of an MRI-based decision tree to differentiate autoimmune and autoinflammatory inner ear disease from chronic otitis media with sensorineural hearing loss
Source: Sci Rep. 2021 Sep 27;11:19171. doi: 10.1038/s41598-021-98557-w (PMC8476614; doi:10.1038/s41598-021-98557-w)
Supplement: Supplementary file 7 — Supplementary Information. [file 41598_2021_98557_MOESM7_ESM.docx]

**Construction of an MRI-based decision tree to differentiate autoimmune and autoinflammatory inner ear disease from chronic otitis media with sensorineural hearing loss**

**Supplementary Information**

**Table S1. Imaging parameters for temporal bone magnetic resonance imaging**

| **Sequence** | **TR** | **TE** | **FA** | **ETL** | **NSA** | **FOV** | **Acquisition matrix** | **Slice thickness** | **Slice gap** |
| --- | --- | --- | --- | --- | --- | --- | --- | --- | --- |
| **Whole-brain axial T2WI** | 3000 | 80 | 90 | 15 | 1 | 180×220 | 400×364 | 5 | 1 |
| **Whole-brain axial FLAIR** | 9000 | 135 | 90 | 35 | 2 | 174×220 | 340×200 | 5 | 1 |
| **3D heavily T2WI (VISTA)** | 2000 | 258 | 90 | 74 | 1 | 160×160 | 228×228 | 0.7 | 0.35 |
| **2D axial T1WI** | 500 | 10 | 50 | 1 | 1 | 180×180 | 272×218 | 3 | None |
| **3D FLAIR (VISTA)** | 4800 | 220 | 90 | 113 | 2 | 200×200 | 256×256 | 0.8 | None |
| **3D post- T1WI (GRE)** | 9.58 | 3.28 | 8 | 64 | 1 | 200×200 | 256×256 | 1 | None |

TR, repetition time; TE, echo time; FA, flip angle; ETL, echo train length; NSA, number of signals averaged; FOV, field-of-view; T2WI, T2-weighted image; FLAIR, fluid-attenuated inversion recovery image; VISTA, volume isotropic turbo spin-echo acquisition; T1WI, T1-weighted image; GRE, gradient-recalled echo sequence

| **Case No. ID (Gender/Age at diagnosis)** | **Clinical diagnosis** | **NLRP3 variant [NM_001243133.1] DNA source: blood** | **Domain** | **Inheritance pattern** | **ACMG classification (Rules)** |
| --- | --- | --- | --- | --- | --- |
| **Case 1.**  SB333-656  (M/13 years) | CINCA syndrome | c.1985T>C:p.Met662Thr | NAD | *De novo* | Likely Pathogenic  (PS2_Strong, PM2) |
| SB334-662 (F/11 years) | CINCA syndrome | c.1709A>G:p.Tyr570Cys | NAD | *De novo* | Likely Pathogenic  (PS2_Very Strong, PM2) |
| **Case 2.**  SB338-669  (F/42 years) | Nonsyndromic HL (DFNA34) | c.2752C>T: p.Arg918Ter | LRRs** | AD*** | Pathogenic  (PVS1, PM2, PM5) |
| **Case 3.**  SB398-767  (F/38 years) | Possibly nonsyndromic HL (DFNA34) | Not detected* | N/A | N/A | N/A |

**Table S2. Detailed genotypes from subjects with autoinflammatory inner ear diseases**

*Possibly with mosaicism of the *NLRP3* variant. **LRRs, Leucine-rich repeats, ***AD, Autosomal dominant; N/A, not applicable

**Table S3. MRI features in patients with AIED/AID**

| **No.** | **Age/Sex** | **Diagnosis** | **Amount/Laterality of mastoid and middle ear effusion** | **Location of inner ear pathology** | **Laterality of inner ear pathology** | **PreFLAIR hyperintensity in inner ear** | **Presence/Degree of postFLAIR inner ear enhancement** | **PostT1WI inner ear enhancement** | **Cranial nerve enhancement on postT1WI** | **Dural & adjacent soft-tissue enhancement on postT1WI** | **RW sign either on postFLAIR or postT1WI** | **VA enhancement on postFLAIR** |
| --- | --- | --- | --- | --- | --- | --- | --- | --- | --- | --- | --- | --- |
| 1 | 67/F | GPA | Severe/Bilateral | Cochlea, vestibule, semicircular canal | Bilateral | **+** | **+**/Intense | - | - | **+** | **+** | **+** |
| 2 | 81/M | GPA | Moderate/Bilateral | Cochlea | Bilateral | NA | **+**/Mild | **+** | - | **+** | **+** | **+** |
| 3 | 63/F | GPA | Severe/Bilateral | Cochlea, vestibule, semicircular canal | Bilateral | NA | **+**/Intense | **+** | - | **+** | **+** | **+** |
| 4 | 48/F | GPA | Mild/Bilateral | Cochlea, vestibule, semicircular canal | Bilateral | **+** | **+**/Intense | **+** | - | **+** | **+** | - |
| 5 | 81/F | GPA | Severe/Bilateral | Cochlea, vestibule | Bilateral | NA | NA | **+** | - | **+** | **+** | NA |
| 6 | 66/M | GPA | Moderate/Bilateral | Cochlea, vestibule | Bilateral | **+** | **+**/Mild | **+** | - | **+** | **+** | **+** |
| 7 | 78/F | GPA | Moderate/Bilateral | Cochlea, vestibule, semicircular canal | Bilateral | NA | **+**/Intense | **+** | - | **+** | **+** | - |
| 8 | 61/F | GPA | Severe/Unilateral | Cochlea, vestibule, semicircular canal | Unilateral | NA | **+**/Intense | **+** | **+** | **+** | **+** | **+** |
| 9 | 67/F | GPA | Severe/Bilateral | Cochlea, vestibule | Bilateral | NA | NA | **+** | - | **+** | - | NA |
| 10 | 77/M | GPA | Mild/Unilateral | Cochlea | Bilateral | NA | **+**/Minimal | - | - | - | **+** | - |
| 11 | 81/M | GPA | Moderate/Bilateral | Cochlea, vestibule, semicircular canal | Bilateral | NA | **+**/Intense | **+** | - | **+** | **+** | **+** |
| 12 | 40/M | Cogan syndrome | None | Cochlea, vestibule, semicircular canal | Bilateral | **+** | **+**/Intense | **+** | - | - | - | **+** |
| 13 | 63/M | Cogan syndrome | Moderate/Bilateral | Cochlea, vestibule, semicircular canal | Bilateral | **+** | **+**/Intense | **+** | - | **+** | **+** | **+** |
| 14 | 7/M | Cogan syndrome | None | Cochlea | Bilateral | **+** | **+**/Intense | - | - | - | **+** | - |
| 15 | 42/F | CINCA syndrome | None | Cochlea, vestibule, semicircular canal | Bilateral | **+** | **+**/Intense | - | - | - | - | **+** |
| 16 | 38/F | CINCA syndrome | None | Cochlea | Bilateral | NA | **+**/Minimal | - | - | - | - | - |
| 17 | 11/F | CINCA syndrome | None | Cochlea, vestibule, semicircular canal | Bilateral | NA | **+**/Intense | - | **+** | - | - | - |
| 18 | 13/M | CINCA syndrome | Mild/Bilateral | Cochlea, vestibule, semicircular canal | Bilateral | NA | **+**/Intense | - | **+** | **+** | - | - |

+, present finding; -, absent finding

MRI, magnetic resonance imaging; AIED/AID, autoimmune/autoinflammatory inner ear disease; GPA, granulomatosis with polyangiitis; CINCA syndrome, chronic infantile neurological, cutaneous, and articular syndrome; preFLAIR, pre-contrast FLAIR image; postFLAIR, post-contrast FLAIR image; postT1WI, post-contrast T1-weighted image; RW sign, round window sign; VA, vestibular aqueduct; NA, not applicable

**Supplementary Figure Legends**

**Figure S1. The degree of the effusion or mastoid and middle ear effusion.** (a) none, (b) mild (arrow), (c) moderate (arrow), and (d) severe (arrow) degree of mastoid effusion.

**Figure S2. Hyperintensity on pre-contrast fluid-attenuated inversion recovery image (preFLAIR).** PreFLAIR-hyperintensities are shown in cochlea and semicircular canal (arrows).

**Figure S3. The degree of inner ear enhancement on post-contrast fluid-attenuated inversion recovery image (postFLAIR).** (a) none, (b) minimal (arrows), (c) mild (arrow), and (d) intense (arrows) enhancements.

**Figure S4. Examples of contrast-enhancement on post-contrast fluid-attenuated inversion recovery image (postFLAIR) and post-contrast T1-weighted image (postT1WI).** Contrast-enhancement is seen in the (a) cochlea (arrow), (b) dura (arrow), (c) cranial nerve (arrows), and (d) nasopharynx (arrow).

**Figure S5. Round window (RW) sign.** On both (a) postFLAIR and (b) postT1WI, focal nodular contrast-enhancement is noted at RW level (arrows).

**Figure S6. Vestibular aqueduct (VA) enhancement.** On postFLAIR, linear contrast-enhancement is seen at bilateral VA (arrows).
